# Identification of quantitative trait loci for increased α-tocopherol biosynthesis in wild soybean using a high-density genetic map

**DOI:** 10.1186/s12870-019-2117-z

**Published:** 2019-11-21

**Authors:** Cheolwoo Park, Maria Stefanie Dwiyanti, Atsushi J. Nagano, Baohui Liu, Tetsuya Yamada, Jun Abe

**Affiliations:** 1 Graduate School of Agriculture, Hokkaido University, Sapporo, 060-8589 Japan; 2 Research Faculty of Agriculture, Hokkaido University, Sapporo, 060-8589 Japan; 3 Faculty of Agriculture, Ryukoku University, Otsu, 520-2194 Japan; 4 School of Life Science, Guangzhou University, Guangzhou, 510000 China

**Keywords:** Functional food, *Glycine max*, Quantitative trait loci, Soybean, Tocopherol, Vitamin E, Wild germplasm

## Abstract

**Background:**

Soybean is one of the most important crop sources of tocopherols (Toc). However, the content of α-Toc, an isoform with the highest vitamin E activity in humans, is low in most cultivars. With the aim of broadening genetic variability, we performed quantitative trait locus (QTL) analysis for a high seed α-Toc trait detected in a wild soybean and characterized the sequence polymorphisms and expression profiles of γ-tocopherol methyltransferase (*γ-TMT*) genes as potential candidates.

**Results:**

A recombinant inbred line population was developed from a cross between the low α-Toc breeding line TK780 and the high α-Toc wild accession B04009. The α-Toc content in seeds correlated strongly with the ratio of α-Toc to γ-Toc contents. QTL analysis using a high-density map constructed with 7710 single nucleotide polymorphisms (SNPs) generated by restriction site-associated DNA sequencing detected six QTLs involved in α-Toc biosynthesis. Of these, three in chromosomes (Chr) 9, 11, and 12 produced consistent effects during a 2-year trial. B04009 allele at QTLs in Chr9 and Chr12 and TK780 allele at the QTL in Chr11 each promoted the conversion of γ*-*Toc to α-Toc, which elevated the seed α-Toc content. SNPs and indels were detected between the parents in three *γ-TMT* genes (*γ-TMT1*, *γ-TMT2,* and *γ-TMT3*) co-located in the QTLs in Chr9 and Chr12, of which some existed in the cis-regulatory elements associated with seed development and functions. In immature cotyledons, *γ-TMT3* was expressed at higher levels in B04009 than TK780, irrespective of two thermal conditions tested, whereas the expression of *γ-TMT*2 was markedly upregulated under higher temperatures, particularly in B04009.

**Conclusions:**

We identified QTLs consistently controlling α-Toc biosynthesis in wild soybean seeds in 2-year trials. The QTL on Chr9 had been previously identified in soybean, whereas the QTLs on Chr11 and Chr12 were novel. Further molecular dissections and characterization of the QTLs may facilitate the use of high α-Toc alleles from wild soybean in soybean breeding and an understanding of the molecular mechanisms underlying α-Toc biosynthesis in soybean seeds.

## Background

Tocopherols (the vitamin E family) are lipophilic antioxidants that prevent the oxidation of unsaturated fatty acids. There are four isoforms of tocopherols, α-, β-, γ-, and δ-tocopherol, of which α-tocopherol (α-Toc) has the highest vitamin E activity in humans because of its highest affinity with the hepatic tocopherol transfer protein [1, 2]. As well as vitamin E activity, α-Toc also plays a role in the prevention of aging-related diseases such as cardiovascular diseases and cancer [1, 2].

Soybean (*Glycine max* Merr.) is one of the most important agricultural crops worldwide because it is a major source of oil, protein, starch, dietary fiber, minerals, and vitamins, and is used as a material in the production of biodiesel, feed, and cosmetics. Soybean oil has a relatively high total tocopherol content compared with other oilseed crops, and the most predominant form is γ-Toc. The α-Toc content is typically less than 10% that of the total Toc content [1, 2]. Considering that soybean is a major oil source providing 30% of the total worldwide oil consumption, increasing the seed α-Toc content may open up opportunities for new food and industrial uses of soybean.

**Fig. 1:**
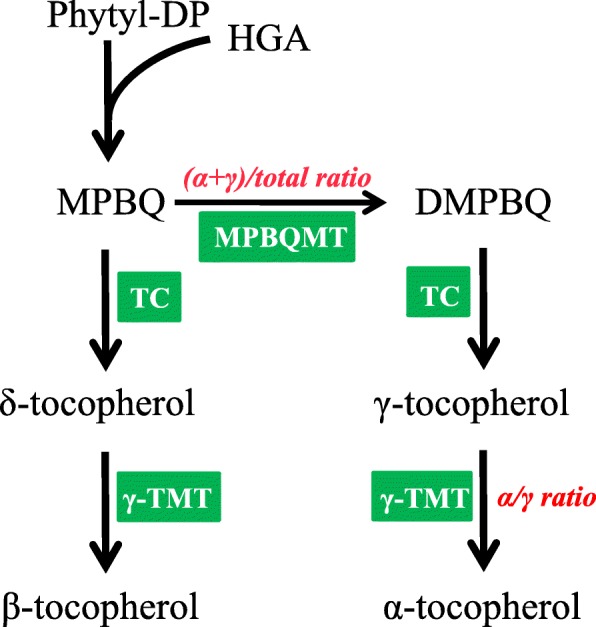
Tocopherol biosynthesis pathway. Enzymes are shown in green boxes. Phytyl-DP, phytyl-diphosphate; HGA, homogentisic acid; MPBQ, 2-methyl-6-phytyl-1,4-benzoquinol; DMPBQ, 2,3-dimethyl-6-phytyl-1,4-benzoquinol; MPBQMT, 2-methyl-6-phytyl-1,4-benzoquinol methyltransferase; TC, tocopherol cyclase; γ-TMT, γ*-*tocopherol methyltransferase

Tocopherol biosynthesis in plants is well characterized [3, 4] (Fig. 1). Fusion between the aromatic head of homogentisic acid (HGA) and the polyprenyl side chain of homogentisate phytyltransferase creates 2-methyl-6-phytyl-1,4-benzoquinol (MPBQ) which is further methylated by MPBQ methyltransferase (MPBQ-MT) to 2,3-dimethyl-6-phytyl-1,4-benzoquinone (DMPBQ). MPBQ and DMPBQ are converted to γ-Toc and δ-Toc, respectively, through cyclization of the HGA head by tocopherol cyclase. The final step in the tocopherol biosynthesis pathway is the conversion of γ-Toc and δ-Toc to α-Toc and β-Toc, respectively, by γ-tocopherol methyltransferase (γ-TMT). MPBQ-MT and γ-TMT are crucial in determining the seed tocopherol composition. γ-TMT activity is reflected in α-Toc/γ-Toc ratio, whereas MPBQ-MT activity is reflected in (α-Toc + γ-Toc)/total Toc ratio (Fig. 1). In *Arabidopsis*, MPBQ-MT and γ-TMT are encoded by *VTE3* and *VTE4*, respectively [5, 6]. The *VTE4* overexpression in soybean seeds was reported to increase the α-Toc ratio by up to 70% [5]. *VTE3* and *VTE4* co-expression further increased the α-Toc ratio by up to 90% and decreased both the δ-Toc and γ-Toc ratios in soybean seeds [5].

Soybean cultivars have variable seed Toc contents and compositions [7–10], and the genetic and molecular bases underlying this natural variation have been extensively studied [11–15]. Dwiyanti et al. (2011) [11] detected a major quantitative trait locus (QTL) in chromosome (Chr) 9 which accounted for 55% of the phenotypic variation in a recombinant inbred line (RIL) population of a cross between the Japanese standard soybean cultivar Ichihime (α-Toc ratio < 10%) and a high α-Toc cultivar from Eastern Europe, Keszthelyi Aproszemu Sarga (KAS; α-Toc ratio > 20%). The QTL region contained a γ-tocopherol methyltransferase gene designated as *γ-TMT3*, which showed higher expression in developing seeds from KAS RILs than those from Ichihime. A β-glucuronidase reporter-aided analysis of *γ-TMT3* further confirmed that the promoter from KAS had higher activity than that from Ichihime, likely caused by single nucleotide polymorphisms (SNPs) in cis-regulatory elements, and MYBCORE and CAAT box motifs in the promoter [11]. Based on these results, Dwiyanti et al. (2011) [11] suggested that the use of *γ-TMT3* with high promoter activity from KAS could be a means of improving the α-Toc content in soybean seeds. The function of γ-TMT3 as a methyltransferase for γ-Toc was confirmed by the catalytic assay of purified enzyme heterologously expressed in *Escherichia coli* [16].

Soybean has additional two tightly-linked *γ-TMT* genes, *γ-TMT1* and *γ-TMT2*, on Chr12 [11]. The three TMT proteins (γ-TMT1, γ-TMT2, and γ-TMT3) exhibit high amino acid similarities of 90.5–94.4% with each other. Based on plastid transit peptide prediction, only γ-TMT2 possesses a plastid transit peptide signal [11]. *γ-TMT2* expression was reported to increase the seed α-Toc content 3–4.5 fold and 4–6 fold when overexpressed in maize (*Zea mays*) and *Arabidopsis*, respectively [17]. It may therefore also be involved in soybean seed α-Toc biosynthesis.

The wild soybean (*Glycine soja*) is a huge reservoir of potentially useful variants for the improvement of soybean cultivars. To date, it has been used to improve yield, stress tolerances, disease resistances, and nutritional components of seeds in soybean breeding [18–26]. Based on a survey on 528 wild soybean accessions collected from various regions of Japan and South Korea, Dwiyanti et al. (2016) [26] discovered 11 accessions with high α-Toc ratios. Sequencing analyses of the promoter and 5′-untranslated region of *γ-TMT3* classified the 11 accessions into four haplotypes, of which one was identical to the *γ-TMT3* sequence of KAS. A molecular genetic study of the high α-Toc ratio of wild accessions, particularly those with novel *γ-TMT3* promoter haplotypes, may therefore be useful in broadening the genetic diversity of α-Toc biosynthesis in soybean.

Here, we report the results of QTL analysis for a high α-Toc trait detected in wild accession B04009, and sequencing and expression analyses for *γ-TMT* genes as potential QTL candidates. The aims of the present study were first to determine whether the elevated α-Toc ratio in B04009 was controlled by the same QTL as detected in the cross with KAS, and second to discover novel genes to improve the seed α-Toc contents of wild accessions.

## Results

### Tocopherol contents and ratios of parental soybean lines under different thermal conditions

Toc contents and compositions were compared between seeds matured at 20 °C and 30 °C, and TK780 and B04009 were shown to have different seed Toc biosynthesis characteristics. TK780 produced seeds with tocopherol contents approximately two-fold higher than B04009, irrespective of the temperature during seed development (Table 1). B04009 produced more α-Toc than TK780 both in 20 °C (*t* = 8.36, *p* = 1.6 × 10^− 4^) and in 30 °C (*t* = 5.71, *p* = 1.3 × 10^− 3^) despite of its lower total Toc content. The α-Toc content elevation in B04009 was associated with increments of both the (α + γ)/total ratio (ratio of the sum of α-Toc and γ-Toc contents to the total Toc content) and the α/γ ratio (ratio of the α-Toc content to the γ-Toc content), reflecting the extent of conversions from MPBQ to DMPBQ and from γ-Toc to α-Toc, respectively. The α-Toc contents increased as temperatures increased in both B04009 (*t* = 7.57, *p* = 1.3 × 10^− 4^) and TK780 (*t* = 3.80, *p* = 0.032), as reported in previous studies [26–32]. The (α + γ)/total and α/γ ratios also increased as temperatures rose, and the increase in the α/γ ratio of B04009, which increased 5.5-fold more at 30 °C than at 20 °C, was particularly marked (Table 1).

**Table 1:** Tocopherol contents and ratios in seeds of TK780 and B04009 produced in different thermal conditions

	Thermal condition	Content (μg/g)				Ratio (%)	
		δ-Toc	γ-Toc	α-Toc	total-Toc	(α + γ)/total ratio	α/γ ratio
TK780	20 °C	178.0 ± 29	204 ± 36	16 ± 8	397 ± 73	55.1 ± 0.9	7.5 ± 2.5
	30 °C	115 ± 4	313 ± 26	43 ± 11	471 ± 20	75.5 ± 0.4	13.8 ± 4.8
B04009	20 °C	63 ± 9	146 ± 38	26 ± 7	236 ± 55	72.8 ± 2.3	17.0 ± 0.9
	30 °C	29 ± 8	82 ± 21	77 ± 18	187 ± 25	84.5 ± 4.1	114.8 ± 15.0

(α + γ)/total ratio = ratio of sum of α-Toc and γ-Toc contents to total-Toc content (%)

α/γ ratio = ratio of α-Toc content to γ-Toc content (%)

### Variation of α-tocopherol contents and ratios in RIL populations

Ninety-four RILs were developed by a single-seed descent method from the F_2_ population of the cross between TK780 and B04009. The two parents differed in flowering habits; under natural daylength (ND) conditions of Sapporo (43°07′N, 141°35′E), TK780 flowered in the middle of July whereas B04009 flowered in late September. The flowering time of RILs also varied widely within the range of parents’ flowering time under ND conditions (data not shown). To reduce the variation induced by different thermal conditions associated with flowering and maturing times, the RILs were grown under short-day conditions in a greenhouse where the air temperature was controlled at 25 °C.

**Fig. 2:**
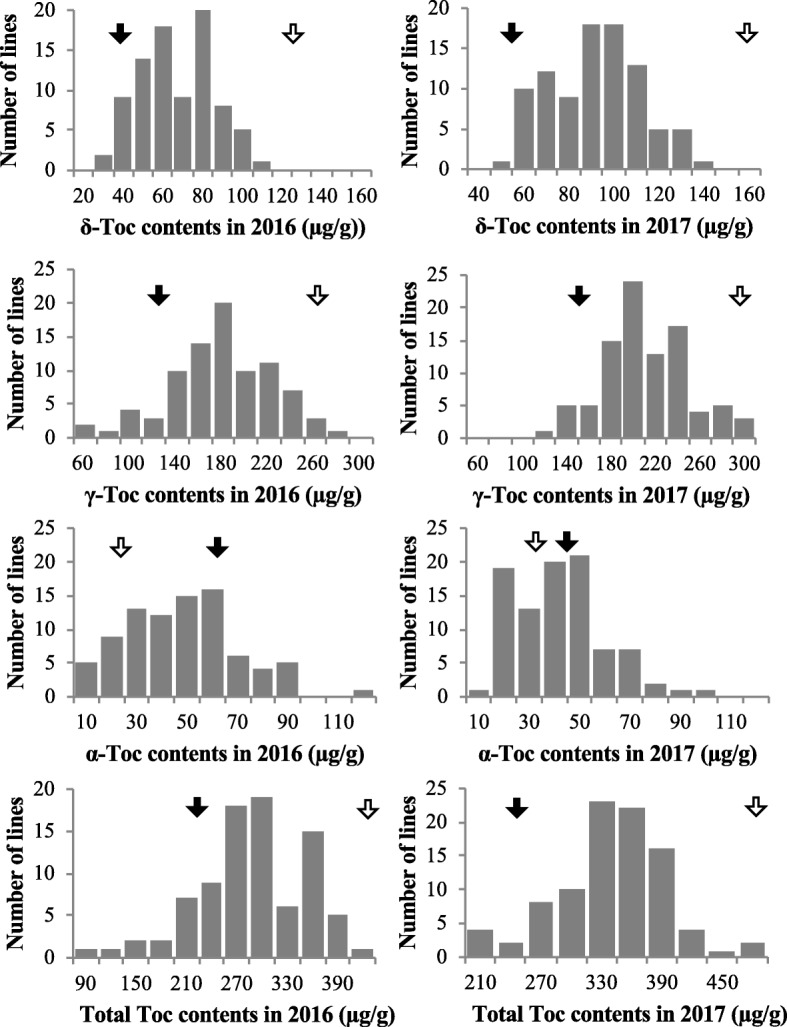
Tocopherol content variation in seeds in RILs of the cross between TK780 and B04009. Closed arrow: B04009, Open arrow: TK780.

**Fig. 3:**
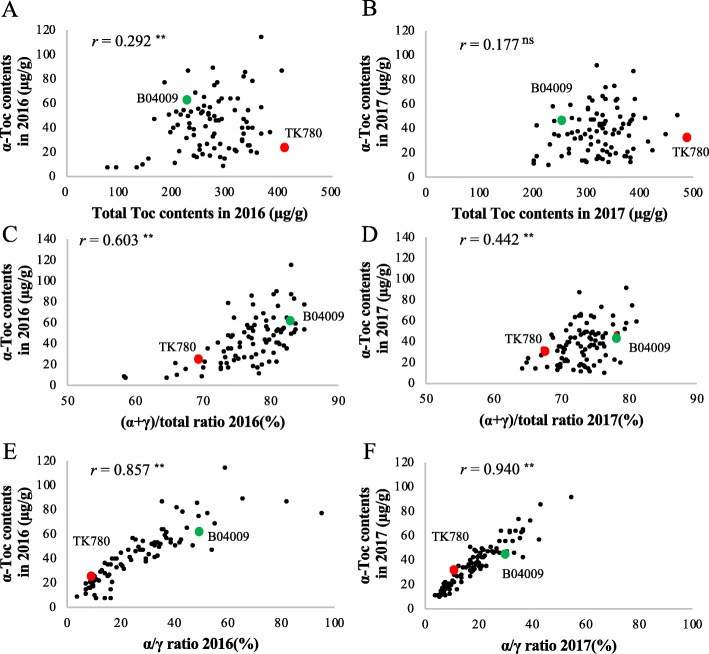
Scatter diagrams showing relationship between seed α-Toc contents with total tocopherol (Toc) contents and Toc ratios in RILs of the cross between TK780 and B04009. Correlations between the seed α-Toc content and the total Toc contents in 2016 (**a**) and in 2017 (**b**). Correlations between the seed α-Toc contents and the (α + γ)/δ-Toc ratios in 2016 (**c**) and 2017 (**d**). The (α + γ)/δ-Toc ratio is the ratio of the sum of α- and γ-Toc contents to the total δ-Toc content. Correlations between the seed α-Toc content and the α/γ-Toc ratios in 2016 (**e**) and 2017 (**f**). The α/γ-Toc ratio is the ratio of the α-Toc content to the γ-Toc content. **, *P* < 0.01; ns, non-significant.

The seed Toc contents in the RILs varied continuously in a range of parental values in δ-Toc, γ-Toc, and total Toc contents, but the α-Toc content was slightly over the parental values in the 2 yrs tested (2016 and 2017) (Fig. 2). The α-Toc contents ranged from 7 to 115 μg/g in 2016 (24 μg/g seed in TK780, 62 μg/g seed in B04009) and from 9 to 91 μg/g in 2017 (32 μg/g seed in TK780, 46 μg/g seed in B04009). The correlation coefficients between years were significant in all contents (*P* < 0.01); the highest was seen in the α-Toc content (*r* = 0.772), suggesting that the biosynthesis of α-Toc was relatively stable compared with that of other tocopherol contents (Additional file 1A). The α-Toc contents did not correlate with δ-Toc and γ-Toc contents in either year (Additional file 1B), but weakly correlated with total Toc contents; the correlation coefficient (*r* = 0.292) was significant only in 2016 (*P* < 0.01; Fig. 3a and b). In contrast, there were strong positive correlations (*r* = 0.747 to 0.931) among δ-Toc, γ-Toc, and total Toc contents (Additional file 1B).

**Fig. 4:**
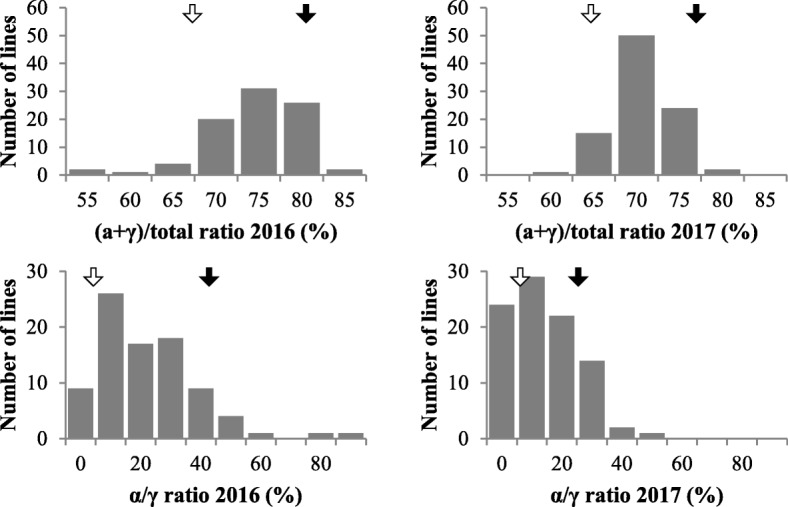
Variation in tocopherol ratios in seeds in RILs of the cross between TK780 and B04009. (α + γ)/total ratio: the ratio of the sum of α- and γ-Toc contents to the total Toc content. α/γ ratio: the ratio of the α-Toc content to the γ-Toc content. Closed arrow: B04009, Open arrow: TK780.

We next surveyed the variation of (α + γ)/total and α/γ ratios in the RIL population. The RILs varied continuously almost in a range of parental values with respect to the (α + γ)/total ratio, whereas some lines showed higher α/γ ratios than B04009 (Fig. 4). Both ratios showed significant (*P* < 0.01) positive correlations between years, and the correlation coefficient was higher in the α/γ ratio (*r* = 0.768) than the (α + γ)/total ratio (*r* = 0.474) (Additional file 1A). Both ratios further positively correlated with the α-Toc content in both years; the correlation with the α-Toc content was stronger for the α/γ ratio than the (α + γ)/total ratio (Fig. 3C to 3F). The two ratios also exhibited significantly positive (P < 0.01) correlations with each other (*r* = 0.541 in 2016, *r* = 0.472 in 2017). Collectively, these results suggest that the seed α-Toc contents in the RIL population were associated primarily with the α/γ ratio, indicating the extent of conversion from γ-Toc to α-Toc, then the (α + γ)/total ratio indicating conversion from MPBQ to DMPBQ, but only weakly with the total tocopherol production (Fig. 4a and b).

### Construction of a high-density linkage map

Prior to QTL analysis, we constructed a genome-wide SNP-based genetic map with a total of 7710 SNPs obtained from restriction site-associated DNA sequencing. Linkage map was constructed using IciMapping software [33]. The entire length of the linkage map was 3211.6 cM, and the length of each chromosome ranged from 121.3 cM for the smallest linkage group of Chr9 to 221.1 cM for the largest one of Chr11. The average genetic distance between neighboring SNP markers was 1.4 cM; the largest gap between SNPs in each chromosome ranged from 5 cM in Chr3 and Chr4 to 34.1 cM in Chr11. The gaps were mostly caused by a lack of SNPs available to map between the two lines.

### QTLs for Toc contents

**Fig. 5:**
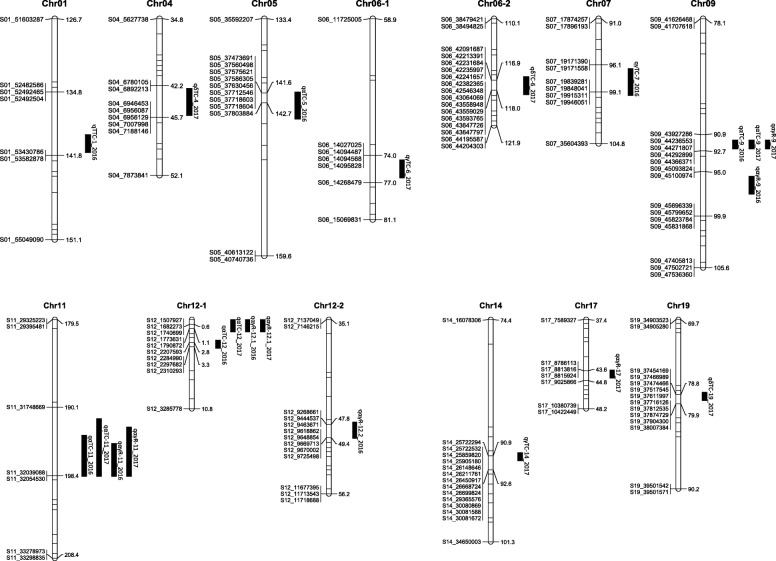
Location of QTLs for tocopherol contents and ratios in RILs of TK780 and B04009 cross

QTL analyses were performed with the inclusive composite interval mapping of additive QTLs implemented in QTL IciMapping [33]. Based on the permutation, a QTL is significant if the logarithm of odds (LOD) score exceeds 3.576 for both years (*P* < 0.05). We detected significant QTLs for the α-Toc content in Chr5 (*qαTC-5*), Chr9 (*qαTC-9*), Chr11 (*qαTC-11*), and Chr12 (*qαTC-12*) in 2016, and in Chr9 (*qαTC-9*) and Chr12 (*qαTC-12*) in 2017 (Table 2 and Fig. 5). Additionally, *qαTC-11* with a LOD score of 3.2 was detected in Chr11 in 2017 although this was below the threshold of significance. The map positions for the QTLs in Chr9, Chr11, and Chr12 were identical or almost the same between the 2 years, suggesting that the effects were caused by identical QTLs. The B04009 allele increased the α-Toc contents at *qαTC-9* and *qαTC-12* but decreased the contents at *qαTC-5* and *qαTC-11*. Of these, *qαTC-9* had the highest LOD scores (14.3 in 2016, 13.1 in 2017) with the largest additive effect on the α-Toc content. Collectively, the QTLs detected accounted for 56.4% (2016) and 54.2% (2017) of the phenotypic variation observed in the RIL population.

**Table 2:** QTLs for seed tocopherol contents in RILs of TK780 and B04009 cross

Trait	Year tested	QTL-Chr	Position (cM)	Support interval (cM)	LOD score	PVE (%)	Additive effect of B04009 allele	Flanking marker^1)^
α-Toc content (μg/g)	2016	*qαTC-5*	142	141.5–144.5	6.1	9.2	−7.6	S05_37473691/ S05_37803884
		*qαTC-9*	92	91.5–92.5	14.3	28.7	12.9	S09_43927286 /S09_44366371
		*qαTC-11*	198	193.5–198.5	4.0	5.8	−5.8	S11_31748669 / S11_32054530
		*qαTC-12*	3	2.5–3.5	7.8	12.7	8.6	S12_2207593/ S12_2310293
	2017	*qαTC-9*	92	91.5–92.5	13.1	39.4	11.2	S09_43927286 / S09_44366371
		*qαTC-11*^1)^	196	191.5–198.5	3.2^2)^	8.4	−5.2	S11_31748669 / S11_32054530
		*qαTC-12*	1	0–1.5	6.0	14.7	6.9	S12_1507927/ S12_1790872
δ-Toc content (μg/g)	2017	*qδTC-4*	43	42.5–45.5	5.5	9.6	6.9	S04_6780105 / S04_7188146
		*qδTC-6*	117	116.5–118.5	4.7	7.9	6.1	S06_42091687 / S06_43647797
		*qδTC-19*	79	78.5–79.5	14.1	32.4	−12.4	S19_37454169 / S19_38007384
γ-Toc content (μg/g)	2016	*qγTC-7*	97	96.5–99.5	4.0	16.4	−19.8	S07_19171390 / S07_19946051
	2017	*qγTC-6*	75	74.5–76.5	5.6	7.3	13.9	S06_14027025 / S06_14268479
		*qγTC-13*	20	16.5–20.5	6.8	8.6	−14.8	S13_14124674/ S13_14460816
		*qγTC-14*	91	90.5–91.5	7.7	9.9	16.1	S14_25722294 / S14_30081672
Total-Toc content (μg/g)	2016	*qTTC-1*	141	139.5–141.5	5.5	22.5	−27.1	S01_52482586 / S01_53582878

1) Physical positions of the QTL support intervals are indicated in the flanking marker names. The two digits after S represent the chromosome, while the number after the underline represents the physical position on the reference genome Williams82.a2

2) *p* = 0.066

PVE = percent of variation explained (%)

QTLs for δ-Toc content were detected only in 2017. Three QTLs (*qδTC-4*, *qδTC-6*, and *qδTC-19*) were detected in the δ-Toc content; the wild allele increased the δ-Toc content at *qδTC-4* and *qδTC-6* and decreased it at *qδTC-19*. Of these, *qδTC-19* exhibited the highest LOD score (14.1), solely accounting for 32% of the phenotypic variation in the RIL population. Three QTLs (*qγTC-6*, *qγTC-13*, and *qγTC-14*) were detected in the γ-Toc content in 2017; the wild allele increased the γ-Toc content at both QTLs, which collectively accounted for 20.2% of the whole variation. One QTL for γ-Toc content in 2016 was detected in Chr7. Only one QTL (*qTTC-1*) was detected in the total-Toc content in 2016. The four QTLs detected in the α-Toc content, therefore, did not overlap with those for the δ-Toc, γ-Toc, and total-Toc contents, consistent with the absence of correlations between the α-Toc content and the other contents (Fig. 3a and b, Additional file 1B).

### QTLs for Toc ratios

QTLs analysis was also performed for (α + γ)/total and α/γ ratios. Significant QTLs were only detected for the α/γ ratio: four QTLs in Chr9 (*qαγR-9*), Chr11 (*qαγR-11*), and Chr12 (*qαγR-12.1* and *qαγR-12.2*) in 2016 and four QTLs in Chr9 (*qαγR-9*), Chr11 (*qαγR-11*), Chr12 (*qαγR-12.1*), and Chr17 (*qαγR-17*) (Table 3, Fig. 5). Because they had identical or almost the same map positions in the 2 years, the three QTLs, *qαγR-9*, *qαγR-11*, and *qαγR-12.1*, consistently controlled the α/γ ratio during this time. The B04009 allele positively controlled the α/γ ratios at *qαγR-9* and *qαγR-12*.*1* but negatively at *qαγR-11*, *qαγR-12.2*, and *qαγR-17*. *qαγR-9* had the highest LOD scores in both years (18.2 in 2016, 21.1 in 2017). Collectively, the four QTLs accounted for 63.3% (2016) and 68.2% (2017) of the whole variation detected in the RIL population. The SNPs flanking *qαγR-9*, *qαγR-11*, and *qαγR-12.1* were identical to or nearby those of QTLs for α-Toc contents (*qαTC-9*, *qαTC-11*, and *qαTC-12*), suggesting that they controlled both the α/γ-Toc ratio and α-Toc contents.

**Table 3:** QTLs for seed tocopherol ratios in RILs of TK780 and B04009 cross

Trait	Year tested	QTL-Chr	Position (cM)	Support interval (cM)	LOD score	PVE (%)	Additive effect of B04009 allele	Flanking marker^1)^
α/γ ratio	2016	*qαγR-9*	96	95.5–97.5	18.2	42.8	6.9	S09_45093824/ S09_45831868
		*qαγR-11*	198	194.5–198.5	5.5	8.5	−3.1	S11_31748669 / S11_32054530
		*qαγR-12.1*	1	0–1.5	5.7	9.8	3.3	S12_1507927 / S12_1790872
		*qαγR-12.2*	49	47.5–49.5	3.7	5.6	−2.5	S12_9268661 / S12_9725498
	2017	*qαγR-9*	92	91.5–92.5	21.1	42.6	4.8	S09_43927286 / S09_44366371
		*qαγR-11*	197	192.5–198.5	4.6	6.3	−1.9	S11_31748669 / S11_32054530
		*qαγR-12.1*	1	0–1.5	10.5	16.0	3.0	S12_1507927 / S12_1790872
		*qαγR-17*	44	43.5–44.5	4.1	5.1	−1.7	S17_8786113 / S17_9025866

1) Physical positions of the QTL support intervals are indicated in the flanking marker names. The two digits after S represent the chromosome, while the number after the underline represents the physical position on the reference genome Williams82.a2

α/γ ratio (%) = ratio of α-Toc content to γ-Toc content

PVE = percent of variation explained (%)

### Additive effects of three QTLs on α/γ ratio

**Fig. 6:**
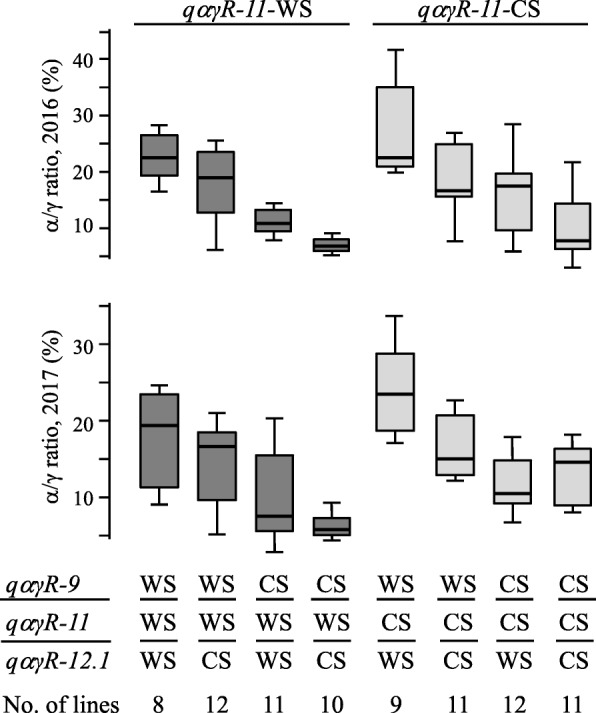
Additive effects of three QTLs for the seed α/γ ratio in RILs of the cross between TK780 and B04009. B04009 and TK780 are designated as WS and CS, respectively

We next evaluated the additive effects of the major three QTLs (*qαγR-9*, *qαγR-11*, and *qαγR-12.1*) for the α/γ ratios. RILs were classified into eight genotypic classes based on the flanking SNPs at the QTLs, and mean α/γ ratios were compared among genotypes. As shown in Fig. 6, alleles from B04009 at *qαγR-9* and *qαγR-12.1* and those from TK780 at *qαγR-11* each additively increased the α/γ ratios in both years.

### Sequence polymorphism of γ-TMT genes as candidates for QTLs for α-Toc biosynthesis

We surveyed the genes annotated in the genomic regions of the QTLs in Chr9, Chr11, and Chr12 for α-Toc contents and α/γ ratios in the Williams 82 genome sequence (Phytozome v12.1/*Glycine max* Wm82.a2.v1). The *qαTC*/*αγR-9* region contained *γ-TMT3* (Glyma.09G22280, physical position: 44,341,974–44,346,311); this was previously identified as a candidate of the QTL for a high α-Toc ratio in the cross between high and low α-Toc soybean cultivars [11]. We identified no gene directly involved in tocopherol biosynthesis between the nearest flanking markers of *qαTC*/*αγR-12.1*, but *γ-TMT2* (Glyma.12G014300.1, physical position: 1,033,151–1,037,054) and *γ-TMT1* (Glyma.12G014200.1, physical position: 1,025,584-1,029,095) were located 703 kb and 711 kb, respectively, apart from the flanking marker S12 1,740,699.

There were 31 genes located within the *qαTC*/*αγR-11* region between markers S11_31748669 and S11_32039088 (Table 2, Additional file 2). Of these, 18 genes are expressed in developing seeds and pods (SoyBase; https://soybase.org/); however, no genes are known to be involved in Toc biosynthesis. The candidate gene may be located in the vicinity of the QTL. Therefore, we investigated the flanking regions and selected approximately 35 genes both upstream and downstream, encompassing a 400-kb region. Among the 100 selected genes (Glyma.11G219000 to Glyma.11G228900), there were four zinc finger transcription factors. Two of these genes are expressed in seeds and pods, RING-H2 FINGER C2A (Glyma.11G220400) and Znf_GATA (Glyma.11G226400) (Additional file 2, SoyBase; https://soybase.org/).

We compared the sequences of three *γ-TMT* genes between TK780 and B04009. The 1350 bp sequence in the promoter region of *γ-TMT3* has already been determined in B04009 and shown to differ with respect to 21 SNPs and four indels from KAS [26]. TK780 and B04009 possessed the same coding sequence as the soybean reference genome Williams 82 (Glyma.09G222800.1), but there were 13 SNPs in the promoter region of which 10 were located within known cis-elements (Additional file 3). TK780 possessed 2SSEEDPROTBANAPA, a cis-element conserved in many storage protein gene promoters [34], and the seed-specific cis-element CANBNNAP [35], whereas B04009 contained MYB1AT and CAATBOX1, which were previously detected as cis-elements specific to cultivars with high α-Toc ratios [11].

A non-synonymous substitution was detected in exon 5 of *γ-TMT2*; the amino acid residue was serine in TK780 in place of threonine in Williams 82 (Glyma.12G014300.1) and B04009 (Additional file 4). A total of 46 DNA polymorphisms, 38 SNPs, and eight indels were detected between the two parents in the promoter and introns of *γ-TMT2* (Additional file 4). Of these, eight polymorphisms were located within known cis-elements: B04009 possessed DRE2COREZMRAB17, a cis-element for genes expressed during late embryogenesis and induced by abscisic acid [36], and SEF4MOTIFGM7S, a cis-element bound by soybean embryo factor 4 [37].

The coding sequences of *γ-TMT1* in both TK780 and B04009 were identical to that of Williams 82 (Glyma.12G014200.1; Additional file 5). Additionally, a total of 17 SNPs and 12 indels were detected in the promoter and introns of *γ-TMT1*, of which seven polymorphisms were located within known cis-elements; there were 13 B04009-specific and four TK780-specific cis-elements, of which PYRIMIDINEBOXHVEPB1 and RYREPEATBNNAPA in B04009 are known to be involved in seed development and function [38].

### Expression profiles for γ-TMT genes in parental lines TK780 and B04009

**Fig. 7:**
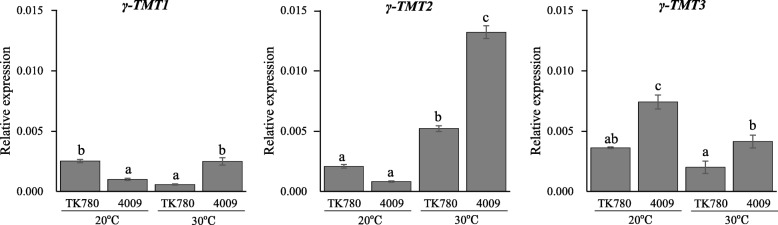
Expression profiles of three *γ-TMT* genes in immature cotyledons matured in 20 °C and 30 °C. The immature cotyledons were obtained from plants grown under 20 °C and 30 °C. The relative expression is presented using *actin* (Glyma.18G222800.1) expression as an internal control. The experiments were repeated four times using independently synthesized cDNAs. Error bars; standard errors, Different alphabets indicate statistically significant (at 5% level) differences between mean values tested by Tukey’s HSD.

Finally, we analyzed the expression levels of *γ-TMT1*, *γ-TMT2*, and *γ-TMT3* in immature cotyledons of full seed size sampled from TK780 and B04009 plants grown in two different thermal conditions after flowering. The expression levels of *γ-TMT1* were significantly higher in TK780 than B04009 at 20 °C, but lower in TK780 than B04009 at 30 °C (Fig. 7). The *γ-TMT2* expression level was lower in B04009 than TK780 at 20 °C, although the difference was not significant. The *γ-TMT2* expression level was significantly upregulated at 30 °C in both lines, although expression was much higher in B04009 than TK780 (Fig. 7). The expression levels of *γ-TMT3* were significantly higher in B04009 than TK780 at both thermal conditions (Fig. 7).

## Discussion

### Three QTLs consistently control α-Toc biosynthesis

QTL mapping and genome-wide association studies (GWAS) have revealed molecular and genetic bases underlying the natural variation in seed tocopherol contents and compositions in *Arabidopsis* [39] and major crops such as maize [40–42], barley [43], rice [44], tomato [45, 46], soybean [11–15], and *Brassica napus* [47–49]. The α-Toc contents and concentrations are strongly associated with *γ-TMT* (*VTE4*) in maize [40, 42] and rice [44]. In a GWAS analysis using 543 maize diverse lines, two insertion/deletions (InDels) within *ZmVTE4* were significantly correlated with α-tocopherol content. One InDels located within the *ZmVTE4* promoter region is associated with the gene expression level [42]. In tomato, epialleles with different DNA methylation levels in the promoter region proximal to the start codon of the gene encoding MPBQ-MT (*VTE3*) affect *VTE3* expression levels and are inversely correlated with γ-Toc contents [46]. As well as those in tocopherol biosynthesis pathways, novel genes also exhibit an association with contents of γ-Toc, δ-Toc, and total Toc, including two genes for protochlorophyllide reductase in chlorophyll biosynthesis and a gene for long-chain acyl-coenzyme A synthase in the fatty acid pathway of maize [40, 41]. Recently, a candidate gene association analysis showed that a 5/8-bp insertion/deletion in promoter region of *ZmPORB2* encoding a protochlorophyllide oxidoreductase is related to total tocopherol content in maize [50].

A number of QTLs for seed α-Toc contents have been reported in soybean [11–15]. Li et al. (2010) [12] identified four QTLs for α-Toc contents by single marker analyses in a cross between the high α-Toc Canadian cultivar OAC Bayfield and the low α-Toc Chinese cultivar Hefeng 25. Shaw et al. (2017) [15] found nine and five QTLs, by single marker analyses and interval mapping, respectively, for α-Toc contents in a cross with OAC Bayfield and a low α-Toc OAC Shire across three locations over 2 yrs, of which the QTL tagged by Satt117 (Chr15) had the largest effect, accounting for up to 32% of the phenotypic variation. Liu et al. (2017) [13] reported a total of 18 QTLs for α-Toc contents in an RIL population of a cross with the Chinese high α-Toc local variety Beifeng 9, of which four QTLs in Chr15 had stable and significant additive effects across six environments. These studies similarly detected QTLs in Chr15, although the candidate genes remained undetermined. However, no genes encoding the enzymes directly involved in α-Toc biosynthesis are colocalized in these QTL regions. Only a QTL detected in a cross with the high α-Toc cultivar KAS harbored *γ-TMT3*; the causal factor in the elevated α-Toc in KAS seeds was considered the higher promoter activity of *γ-TMT3* [11].

In the present study, we identified three QTLs that were consistently detected in a 2-year experiment in the RIL population of a cross between the high α-Toc wild accession B04009 and low α-Toc breeding line. In the RIL population, the α-Toc contents varied in close association with the α/γ ratio, which is the index for conversion efficiency from γ-Toc to α-Toc mediated by γ-TMT; however, the contents of total Toc or other isoforms did not vary in association with the α-Toc content. The three QTLs detected were involved in both the α-Toc content and the α/γ ratio. These QTLs may therefore bring about the accumulation of more α-Toc in seeds by enhancing the conversion from γ-Toc to α-Toc. The QTL with the largest effect was located close to the QTL in Chr9 previously detected in the cross between Ichihime and KAS (Tables 2 and 3, Fig. 5), suggesting that the high α-Toc trait in B04009 may be controlled by the same QTL detected in KAS. Because B04009 and TK780 have an identical *γ-TMT3* coding sequence and the former exhibited higher *γ-TMT3* expression compared with the latter (Additional file 3 and Fig. 7), the QTL may be attributed to different promoter activities of *γ-TMT3* as previously reported [11].

Wild soybean accessions with high seed α-Toc ratios including B04009 exhibited a diverse range of DNA polymorphisms in the promoter sequence (1350 bp) of *γ-TMT3*. B04009 differed with respect to 14 SNPs and four indels from KAS [26]. However, all wild accessions with high α-Toc ratios shared three SNPs with KAS, which were differentiated from the low α-Toc cultivars tested [26]. An investigation of diverse germplasm collections would be useful to confirm the associations of these SNPs with seed α-Toc contents/ratios, and the identification of critical cis-element(s) would aid an understanding of seed α-Toc content control by *γ-TMT3*.

The other two QTLs, *qαγR-11*/*qαTC-11* and *qαγR-12.1*/*qαTC-12*, are novel. The genomic region of *qαγR-12.1*/*qαTC-12* is close to linked pair of *γ-TMT* genes, *γ-TMT1* and *γ-TMT2*, suggesting that either gene is a probable candidate of *qαγR-12.1*/*qαTC-12*. Expression analysis of immature cotyledons developed in different thermal conditions revealed that only *γ-TMT2* of the three *γ-TMT* genes was upregulated by higher temperatures, and that thermal responses differed between the parents (Fig. 7). The elevated α-Toc contents under higher temperatures observed in B04009 were likely caused by its stronger upregulation of *γ-TMT2* expression induced by high temperatures. *γ-TMT2* sequences of the two parents showed a non-synonymous substitution and many DNA polymorphisms in the promoter. Further studies, such as fine mapping, over-expression, and complementary gene studies, are needed to confirm the association of nucleotide variations in *γ-TMT2* with a high α-Toc content.

The QTL in Chr11 had an opposite effect compared with the other two QTLs, and likely contributed to the transgressive segregation in the seed α-Toc contents and α/γ ratios in the RIL population. The genomic region of the QTL contained no genes involved in Toc biosynthesis but included two zinc finger transcriptional factors (Glyma.11G220400 and Glyma.11G226400) expressed in developing seeds. Of these, GATA-type zinc fingers are known GATA-binding transcriptional factors that control embryo development in *Arabidopsis* [51]. Furthermore, the synthetic zinc finger transcriptional factor, fused to a nuclear localization signal and the maize C1 activation domain, successfully upregulated *γ-TMT* by binding to cis-elements in the promoter to elevate the seed α-Toc ratio in *Arabidopsis* [52]. Additional studies, such as a transcriptomics analysis of the two parental lines under low and high temperature regimes, are needed to determine whether these zinc finger transcriptional factors are possible candidates for the QTL and to investigate other candidate genes’ roles in the thermal responses.

### Thermal responses of *γ-TMT* genes in α-Toc biosynthesis during seed development

The temperature during seed development is one of the environmental factors that influence the α-Toc contents and ratios in seeds [53]. α-Toc contents and ratios increase as temperatures rise during seed maturation [26–32]. As expected, the α-Toc contents in seeds of the parental lines used in this study increased as temperatures rose from 20 °C to 30 °C, in parallel with increments of (α + γ)/total and α/γ ratios (Table 1). The increment of α-Toc contents in B04009 was particularly noticeable, and this was most likely caused by the rising α/γ ratio indicating the conversion efficiency from γ-Toc to α-Toc mediated by γ-TMT. The expression of three *γ-TMT* genes in immature cotyledons was controlled by different regulatory systems between the two lines: *γ-TMT3* was expressed at higher levels in B04009 than TK780 irrespective of the two thermal conditions tested, whereas the expression of *γ-TMT*2 was upregulated under the higher temperature in both parents, but the response was particularly marked in B04009 (Fig. 7). Because of the increased accumulation of α-Toc in seeds coincident with the more highly upregulated *γ-TMT2* expression by higher temperatures in B04009, it will be intriguing to confirm whether *γ-TMT2* is responsible for the QTL and if the effect of the B04009 allele is temperature-dependent.

Three *γ-TMT* genes in soybean have high amino acid similarities to each other, except for the N-terminal region in which only *γ-TMT2* possesses a plastid transit peptide signal [11]. RNA-sequencing data deposited in SoyBase (https://soybase.org/) further indicates that the three genes are expressed at relatively lower levels in immature cotyledons during seed development but are variable in transcript abundance in other tissues; compared with other *γ-TMT* genes, the expression of *γ-TMT2* (Glyma.12 g014300) and *γ-TMT3* (Glyma.09 g222800) is upregulated in young leaves and full-size pod shells and roots, respectively [54]. The different protein structures and gene expression profiles and their thermal responses observed in this study may therefore indicate a differentiated role among the three *γ-TMT* genes in the adaptation to oxidative stresses that occur during various phases of development.

Tocopherols are lipophilic antioxidants, and their primary function is to limit non-enzymatic lipid oxidation during seed storage, germination, and early seedling development [55]. A previous genetic study using dysfunctional *VTE2* mutants (*MPBQ-MT*), which lacked all isoforms of tocopherols, demonstrated that tocopherols are essential for seed longevity, germination, and seedling growths [55]. However, different isoforms may have different functions to oxidative stresses.

In tobacco, *γ-TMT*-silenced plants in which α-Toc contents were reduced by up to 95% in leaves showed an elevated susceptibility toward salt stress, but a diminished susceptibility toward osmotic and oxidative stresses from methyl viologen-induced reactive oxygen species, suggesting that γ-Toc is more potent than α-Toc in conferring desiccation tolerance [56]. The β-Toc content is reported to negatively correlate with seed longevity in rice cultivars [57]. Moreover, the overexpression of heterologous *γ-TMT* genes from *Arabidopsis*, *Perilla frutescens*, and *B. napus* by seed-specific promoters in soybean seeds successfully converted γ-Toc to α-Toc to elevate the proportion of α-Toc in the total Toc by up to ≥70% [5, 58–60]. Tavva et al. (2007) [58] found that the increment of α-Toc in seeds of transgenic plants was associated with the reduction of lipid peroxidation products in seeds and germinating seeds, although no specific differences were observed in seed germination or seedling growth between wild-type and transgenic plants. The greater production of α-Toc in seeds concomitant with the upregulation of *γ-TMT2* by higher temperatures might therefore reflect its adaptive function in seed storage, germination, and early seedling development, as reported in *B. napus γ-TMT*-overexpressing soybean plants that produced seeds with more α-Toc to decrease lipid peroxidation products [58].

## Conclusions

We identified two major QTLs and one minor QTL conferring higher α-Toc contents by promoting the conversion of γ-Toc to α-Toc in an RIL population derived from a cross between a wild soybean having a high α-Toc level and a soybean having a low α-Toc level. One QTL containing *γ-TMT3* had been previously identified in soybean, suggesting that *γ-TMT3* controls α-Toc contents in both soybean and wild soybean. The novel QTL in Chr12 is located near *γ-TMT1* and *γ-TMT2.* The incremental increases in the α-Toc contents associated with rising temperature were coincident with the upregulated expression of *γ-TMT2* at high temperatures. The verification of the functions of the three *γ-TMT* genes by genetic transformation will increase our understanding of α*-*Toc biosynthesis and its thermal responses, as well as their genetic diversity in wild and cultivated soybeans. In addition to their contributions to high vitamin E activity, which can improve human health, it is important to characterize the functions of different tocopherol isoforms in plant developmental processes, such as seed development and longevity.

### Methods

### Plant materials

The soybean breeding line TK780 and a wild accession (B04009) were used in this study. TK780 is an early-flowering line with a low seed α-Toc content and ratio. B04009, a wild soybean accession originally from Yamanashi Prefecture, Japan, has a high seed α-Toc content and ratio [26]. The seeds of B04009 were obtained from the Genebank of National.

Agriculture and Food Research Organization (NARO), Tsukuba, Japan. An RIL population of 94 F_8_ (2016) and F_9_ (2017) lines was developed by a single seed descendent method from an F_2_ population of the cross between TK780 and B04009. RILs and parents were grown in a pot under short-day greenhouse conditions where air temperatures were set to 25 °C with a fluctuation from 20 °C° to 30 °C from February to May in 2016 and 2018. Lights were supplied with high intensity discharge lamps (HONDA-T; Panasonic Co., Osaka, Japan) in the daytime. Seeds were harvested as a bulk from three to four plants in each line and dried in a desiccator until required for the assay. Parents were also grown at 25 °C in the greenhouse, and after flowering were transferred to growth chambers set to 20 °C and 30 °C; seeds were harvested individually from two to five plants.

### Tocopherol quantification

Tocopherol contents of seeds were quantified according to Dwiyanti et al. (2011) [11]. Ten fully-dried seeds for each line were ground to a fine powder by Multi-Beads Shocker (MB75 5 U(S); Yasui Kikai Co, Osaka, Japan). Twenty mg of seed powder was thoroughly mixed with 500 μL of cold 80% ethanol (4 °C) containing 5 μL dl-Tocol solution (10 μg/ml; Tama Biochemical Co. Ltd., Tokyo, Japan) as an internal standard. After sonication for 10 min, the mixture was thoroughly mixed with 1000 μL of hexane with added pyrogallol as an antioxidant. The mixture was sonicated again for 10 min, and then centrifuged for 5 min at 18,900×*g* at 4 °C. The supernatants were analyzed by high-performance liquid chromatography (LaChrom Elite, Hitachi High-Technologies Corp., Tokyo, Japan) using a reverse-phase column (Inertsil ODS-3, 3.0 mm × 250 mm; GL Sciences, Tokyo, Japan) with methanol:acetonitrile (10:90 v/v) as a mobile phase at flow rate 0.5 mL/min at a constant temperature of 40 °C. Tocopherol isoforms were detected at 295 nm with one peak for dl-Tocol and three peaks for δ-Toc, the sum of γ-Toc and β-Toc, and α-Toc, in order of retention time. The content of each isoform was calculated with the ratio of the peak area against that of dl-Tocol. In this study, the content calculated from the sum of γ-Toc and β-Toc was considered the γ-Toc content because the actual β-Toc content is very low in soybean seeds [7]. The assay was carried out in triplicate.

### Genotype data generation

Total DNA of RILs was extracted from young leaves using the modified CTAB method [61]. DNA was digested using the restriction enzymes *Bgl*II and *Eco*RI to create a DNA library for double digest restriction site-associated DNA sequencing [62, 63]. Sequencing was performed with 51 bp single-end reads in one lane of a HiSeq2000 Sequencer (Illumina, San Diego, CA, USA) by Macrogen (Seoul, South Korea). The resulting reads were trimmed with Trimmomatic ver 0.33 [64] using the following parameters: LEADING:19, TRAILING:19, SLIDINGWINDOW:30:20, AVGQUAL:20, and MINLEN:51. These RAD-Seq procedures were carried out by Clockmics, Inc. (Izumi, Osaka, Japan). The trimmed reads were mapped to the soybean reference genome Williams82.v2 using Bowtie2 [65] with a default parameter setting. SNP calling was performed using GATK-Unified Genotyper [66]. Imputation of missing genotypes in RILs using parental SNP data was performed using Beagle 4.0 [67]. Filtering for monomorphic SNPs and SNPs having many missing calls was performed using TASSEL.5.2.31 with following parameters: minimum call rate per SNP 90% and minimum allele frequency 0.05 (to remove monomorphic SNPs) TASSEL (v 5.2.31) [68]. Using custom script in R [69], the nucleotide information was converted to AB genotype with parentA = TK780 and parentB=B04009. All heterozygous genotypes were converted as missing alleles. Further filtering for duplicated markers or markers having switch alleles was performed in R/QTL [70] resulting in a final set of 7710 SNPs.

### Linkage group construction and QTL mapping

QTL IciMapping ver 4.1 [33] was used to construct a linkage map with 7710 SNP markers. The Input algorithm, which re-estimates recombination frequency and genetic distance without changing the marker order in the input file, was used to determine the order of markers on the genetic map. The sum of adjacent recombinant frequencies with a window size of 5 was used as a rippling criterion for fine tuning of the markers. Recombination frequencies between linked loci were transformed into centimorgan (cM) distances using Kosambi’s mapping function [71]. Linkage map was drawn using MapChart [72]. Complete linkage map is shown in Additional File 6.

QTL analyses were performed with the inclusive composite interval mapping of additive QTLs implemented in QTL IciMapping [33]. The permutation test was performed to determine the threshold of significant QTLs. Based on the results, LOD scores greater than 3.576 were used as a criterion to delineate the significance levels of QTLs (*P* < 0.05). The supporting intervals of QTLs meeting this threshold were defined by ICIMapping as leftCI, indicating the left border of the confidence interval, and rightCI, indicating the right border of the confidence interval.

### Construction of *γ-TMT* genes based on whole genome resequencing data

Raw reads of TK780 and B04009 from next-generation sequencing Illumina Hiseq XTen were aligned to the soybean reference genome Williams82.a2 [73]. The alignment was performed using Bowtie2–2.2.9 [65]. The resulting alignment was further processed to remove duplicate reads and to fix mate information using Picard tools (http://broadinstitute.github.io/picard). The Genome Analysis Toolkit (GATK ver 3.8 [66];) was used to realign small indels. Subsequently, variants (SNP and indels) were called using the GATK Unified Genotyper function which filtered out reads having mapped base quality Phred scores below 20. Using the reference genome Williams82.a2 and SNP dataset of each variety, sequences of *γ-TMT* genes were reconstructed by the FastaAlternateReferenceMaker function available in GATK.

### Cis-element prediction

New PLACE, a Plant Cis-acting Regulatory DNA Elements database (http://www.dna.affrc.go.jp/PLACE/) [74] was used to predict the position of *cis*-acting regulatory elements in the promoter region (2000-bp upstream of the start codons of *γ-TMT1*, *γ-TMT2*, and *γ-TMT3* of B04009 and TK780.

### RNA extraction and expression analysis

Three to four pods of full seed stage [75] were sampled individually from two to five plants of TK780 and B04009 grown at 20 °C and 30 °C (12 h light/12 h dark). The immature seed samples were immediately frozen in liquid nitrogen and were stored at − 80 °C until RNA extraction. RNA extraction and cDNA synthesis were performed following procedure previously described [76]. Transcript levels of *γ-TMT1*, *γ-TMT2*, and *γ-TMT3* were determined by quantitative real-time PCR using SYBR Premix Ex Taq II (Takara, Japan) and following protocols: 95 °C for 3 min followed by 39 cycles of 95 °C for 10 s, 57 °C for 20 s, 72 °C for 20 s, and 78 °C for 2 s in a CFX96 Real-Time System (Bio-Rad, Osaka, Japan). Primer sequences used in expression analyses are listed in Table 4. The expression levels of three *γ-TMT* genes were normalized against the expression level of Actin (Glyma.18G222800.1). Four independently synthesized cDNAs were used as replications.

**Table 4:** Primer sequences for *γ-TMT* gene expression analysis

Primer Name	Primer Sequence
GTMT-1_F1	5′-CTGGAGGCAGAGTATAGCG-3’
GTMT-1_R1	5′-AAACTCCCAGGTCCCACCCAAT-3’
GTMT-2_F1	5′-GAAGCAAGTTTCCAACAGGTCG-3’
GTMT-2_R1	5′-CGCCAATCATAGGAGATATTGCATATG-3’
GTMT-3_F1	5′-CAGTGGACTTAAAACCATAAAGGGAGC-3’
GTMT-3_R1	5′-CCACATACTCTATATCATTCACACGAG-3’
qGmActin-F	5′-CGGTGGTTCCTATCTTGGCATC-3’
qGmActin-R	5′-GTCTTTCGCTTCAATAACCCTA-3’

### Statistical analysis

Effects of temperatures, parental lines and their interactions on the tocopherol biosynthesis were carried out with two-way analysis of variance. Test of significance among means of transcript abundances was carried out with Tukey’s honestly significant difference (HSD) test.

## Supplementary information

**Additional file 1. **A. Correlation coefficients (*r*) between years for tocopherol contents and ratios. B. Correlation coefficients between contents of tocopherol isoforms and total tocopherol.

**Additional file 2. **Annotated genes in the *qαTC/αγR-11* genomic and flanking regions and their expression levels in seeds and pods. Possible candidate genes are highlighted in yellow, while genes located within *qαTC/αγR-11* are highlighted in grey.

**Additional file 3. **Sequence polymorphisms in *γ-TMT3* gene and promoter region. A. The *γ-TMT3* gene’s structure. Exons are shown as red boxes, and areas between the two exons are introns. The 5′- and 3′-UTRs are shown as grey boxes. Arrows indicate the SNPs and indels located in the cis-elements (C). Numbers above the arrows correspond to the DNA polymorphism numbers in (B). B. All SNPs and indels found in gene and promoter regions between TK780 and B04009. The third row shows the positions of nucleotide polymorphisms relative to the translational start site (ATG). C. List of SNPs and indels located within known cis-elements based on the New PLACE prediction. Cis-elements present in TK780 but not in B04009 are shown in black, whereas cis-elements present in B04009 but not in TK780 are shown in red. SNP or indel positions are underlined in the sequence column. Cis-elements specific to seeds are written in bold letters.

**Additional file 4. **Sequence polymorphisms in *γ-TMT2* gene and promoter region. A. *γ-TMT2* gene structure is depicted as follows. Exons are shown as red boxes, and areas between two exons are introns. The 5′-UTR and 3′-UTR are shown as grey boxes. Arrows shows all SNPs and indels located in cis-elements (C). Numbers above arrows correspond to DNA polymorphism numbers in (B). B. All SNPs and indels found in gene and promoter region between TK780 and B04009. Third row shows the position of nucleotide polymorphisms relative to translational start site (ATG). C. List of SNPs and indels located within known cis-elements based on the New PLACE prediction. Cis-elements present in TK780 but not in B04009 are shown in black, whereas cis-elements present in B04009 but not in TK780 are shown in red. SNPs or indels position are underlined in Sequence column. Cis-elements specific to seed are written in bold letters.

**Additional file 5. **Sequence polymorphisms in *γ-TMT1* gene and promoter region. A. *γ-TMT1* gene structure is depicted as follows. Exons are shown as red boxes, and areas between two exons are introns. The 5′-UTR and 3′-UTR are shown as grey boxes. Arrows shows all SNPs and indels located in cis-elements (C). Numbers above arrows correspond to DNA polymorphism numbers in (B). B. All SNPs and indels found in gene and promoter region between TK780 and B04009. Third row shows the position of nucleotide polymorphisms relative to translational start site (ATG). C. List of SNPs and indels located within known cis-elements based on the New PLACE prediction. Cis-elements present in TK780 but not in B04009 are shown in black, whereas cis-elements present in B04009 but not in TK780 are shown in red. SNPs or indels position are underlined in Sequence column. Cis-elements specific to seed are written in bold letters.

**Additional file 6.** The ‘TK780’ × ‘B04009’ linkage map. The map was created based on 7710 SNPs genotypes generated by restriction-site associated DNA sequencing.

## Data Availability

The datasets used and/or analyzed during the current study are available from the corresponding author on request.
