# Supplementary material for: Identification of quantitative trait loci for increased α-tocopherol biosynthesis in wild soybean using a high-density genetic map
Source: BMC Plant Biol. 2019 Nov 21;19:510. doi: 10.1186/s12870-019-2117-z (PMC6873731; doi:10.1186/s12870-019-2117-z)
Supplement: Supplementary file 3 — Additional file 3. Sequence polymorphisms in γ-TMT3 gene and promoter region. A. The γ-TMT3 gene’s structure. Exons are shown as red boxes, and areas between the two exons are introns. The 5′- and 3′-UTRs are shown as grey boxes. Arrows indicate the SNPs and indels located in the cis-elements (C). Numbers above the arrows correspond to the DNA polymorphism numbers in (B). B. All SNPs and indels found in gene and promoter regions between TK780 and B04009. The third row shows the positions of nucleotide polymorphisms relative to the translational start site (ATG). C. List of SNPs and indels located within known cis-elements based on the New PLACE prediction. Cis-elements present in TK780 but not in B04009 are shown in black, whereas cis-elements present in B04009 but not in TK780 are shown in red. SNP or indel positions are underlined in the sequence column. Cis-elements specific to seeds are written in bold letters. [file 12870_2019_2117_MOESM3_ESM.pdf]

A.

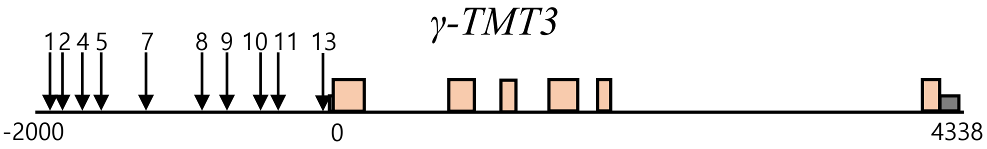

B.

Glyma.09G222800.1

	1	2	3	4	5	6	7	8	9	10	11	12	13
	Promoter												
	-1912	-1818	-1745	-1680	-1551	-1406	-1266	-865	-692	-467	-338	-147	-45
W82	G	G	G	T	A	A	C	G	A	A	A	T	C
TK780	G	G	G	C	A	A	C	G	A	A	A	T	C
B04009	A	A	A	T	C	G	T	A	G	T	C	C	A

	14	15
	Intron1	Intron2
	360	1118
W82	C	A
TK780	G	G
B04009	C	A

C.

No	Cis-elements	Sequence	Strand
1	SITEIIATCYTC	TGGGCY	+
2	GATABOX<br>**DPBFCOREDCDC3**	GATA<br>ACACNNG	+<br>+
4	**2SSEEDPROTBANAPA**<br>**CANBNNAPA**	CAAACAC<br>CNAACAC	+<br>+
5	GT1CONSENSUS<br>**SORLIP1AT**	GRWAAW<br>GCCAC	+<br>-
7	**DPBFCOREDCDC3**	ACACNNG	-
8	CACTFTPPCA1<br>GTGANTG10	YACT<br>GTGA	-<br>+
9	ROOTMOTIFTAPOX1	ATATT	-
10	MARTBOX	TTWTWTTWTT	-
11	TATABOX5<br>GTGANTG10	TTATTT<br>GTGA	-<br>-
13	**MYB1AT**<br>REALPHALGLHCB21<br>**CCAATBOX1**<br>**CAATBOX1**<br>ANAERO1CONSENSUS	WAACCA<br>AACCAA<br>CCAAT<br>CAAT<br>AAACAAA	+<br>+<br>+<br>+<br>+
