# Supplementary material for: Identification of quantitative trait loci for increased α-tocopherol biosynthesis in wild soybean using a high-density genetic map
Source: BMC Plant Biol. 2019 Nov 21;19:510. doi: 10.1186/s12870-019-2117-z (PMC6873731; doi:10.1186/s12870-019-2117-z)
Supplement: Supplementary file 4 — Additional file 4. Sequence polymorphisms in γ-TMT2 gene and promoter region. A. γ-TMT2 gene structure is depicted as follows. Exons are shown as red boxes, and areas between two exons are introns. The 5′-UTR and 3′-UTR are shown as grey boxes. Arrows shows all SNPs and indels located in cis-elements (C). Numbers above arrows correspond to DNA polymorphism numbers in (B). B. All SNPs and indels found in gene and promoter region between TK780 and B04009. Third row shows the position of nucleotide polymorphisms relative to translational start site (ATG). C. List of SNPs and indels located within known cis-elements based on the New PLACE prediction. Cis-elements present in TK780 but not in B04009 are shown in black, whereas cis-elements present in B04009 but not in TK780 are shown in red. SNPs or indels position are underlined in Sequence column. Cis-elements specific to seed are written in bold letters. [file 12870_2019_2117_MOESM4_ESM.pdf]

A.

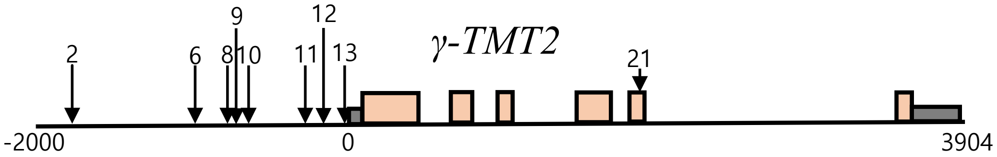

B. Glyma.12G014300.1

	1	2	3	4	5	6	7	8	9	10	11	12	13
	Promoter												
	-1797	-1790	-1281	-1200	-1019	-1017	-862	-791	-728	-691	-296	-179	-30
W82	C	A	-	-	-	TATATTTTATACCGACTGG	C	T	G	T	A	T	A
TK780	A	C	T	AA	-	-	A	A	G	C	T	T	A
B04009	C	A	-	-	GG	TATATTTTATACCGACTGG	C	T	T	T	A	A	T

	14	15	16	17	18	19	20	21
	Intron1		Intron3		Exon4	Intron4		Exon5
	432	515	1153	1267	1480	1694	1698	1870
W82	G	A	G	C	C	-	ATTT	A
TK780	G	A	G	T	T	AT	ATTT	T
B04009	T	G	A	C	C	-	-	A

	Exon5
TK780	GLSSLLSS
B04009	GLTSLLSS

	22	23	24	25	26	27	28	29	30	31	32	33	34	35	36	37	38	39	40	41	42	43	44	45	46
	Intron5																						3'UTR		
	2723	2724	2731	2795	2817	3063	3081	3086	3087	3103	3138	3142	3168	3181	3192	3230	3247	3267	3271	3274	3277	3322	3641	3814	3856
W82	T	A	T	T	A	A	T	T	C	G	C	C	G	C	A	T	A	T	T	C	-	A	G	A	G
TK780	A	A	T	C	A	G	G	G	G	A	C	T	T	T	G	T	G	T	T	C	-	-	C	C	T
B04009	T	T	C	T	T	A	T	T	C	G	G	C	G	C	A	C	A	G	A	A	AGAAC	A	G	A	G

C.

No.	Cis-elements	Sequence	Strand
2	TATABOX5	AAATAA(TTATTT)	-
6	LECPLEACS2<br>ROOTMOTIFTAPOX1<br>DRE2COREZMRAB17<br>DRECRTCOREAT<br>CBFHV<br>LTRECOREATCOR15(T)	TAAAATAT<br>ATATT<br>ACCGAC<br>RCCGAC<br>RYCGAC<br>CCGAC	-<br>+<br>+<br>+<br>+<br>+
8	INRNTPSADB	YTCANTYY	-
9	GATABOX<br>IBOXCORE<br>OSE2ROOTNODULE<br>POLLEN1LELAT52	GATA<br>GATAA<br>CTCTT<br>AGAAA	+<br>+<br>-<br>+
10	BIHD10S	TGTCA	-
11	ARR1AT	NGATT	+
12	S1FBOXSORPS1L21	ATGGTA	+
13	ROOTMOTIFTAPOX1<br>-10PEHVPSBD<br>SEF4MOTIFGM7S	ATATT<br>TATTCT<br>RTTTTTR	+<br>+<br>-
