# Supplementary material for: Identification of quantitative trait loci for increased α-tocopherol biosynthesis in wild soybean using a high-density genetic map
Source: BMC Plant Biol. 2019 Nov 21;19:510. doi: 10.1186/s12870-019-2117-z (PMC6873731; doi:10.1186/s12870-019-2117-z)
Supplement: Supplementary file 5 — Additional file 5. Sequence polymorphisms in γ-TMT1 gene and promoter region. A. γ-TMT1 gene structure is depicted as follows. Exons are shown as red boxes, and areas between two exons are introns. The 5′-UTR and 3′-UTR are shown as grey boxes. Arrows shows all SNPs and indels located in cis-elements (C). Numbers above arrows correspond to DNA polymorphism numbers in (B). B. All SNPs and indels found in gene and promoter region between TK780 and B04009. Third row shows the position of nucleotide polymorphisms relative to translational start site (ATG). C. List of SNPs and indels located within known cis-elements based on the New PLACE prediction. Cis-elements present in TK780 but not in B04009 are shown in black, whereas cis-elements present in B04009 but not in TK780 are shown in red. SNPs or indels position are underlined in Sequence column. Cis-elements specific to seed are written in bold letters. [file 12870_2019_2117_MOESM5_ESM.pdf]

γ-TMT1

A.

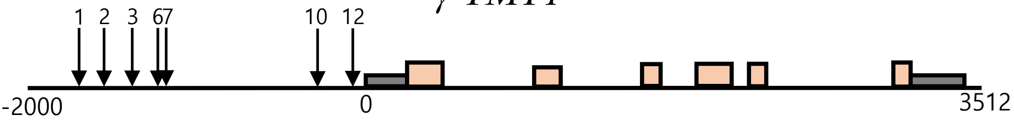

B.

Glyma.12G014200.1

	1	2	3	4	5	6	7	8	9	10	11	12
	Promoter											
	-1697	-1561	-1395	-1317	-1241	-1231	-1192	-1188	-452	-307	-296	-95
W82	T	A	-	-	G	G	A	-	-	GACAA	TT	G
TK780	T	G	-	-	G	C	A	-	T	-	-	A
B04009	C	A	AATA	T	T	G	T	T	-	GACAA	TT	G

	13	14	15	16	17	18	19	20	21	22	23	24	25	26	27	28	29	30
	Intron1						Intron2				Intron3	Intron4					Intron5	
	478	499	631	697	709	789	1246	1406	1413	1509	1937	2760	2801	2813	2976	3011	3301	3311
W82	A	T	T	-	A	T	T	T	A	C	C	A	C	A	C	G	A	A
TK780	A	T	T	-	A	T	A	T	-	C	C	A	A	-	C	G	G	-
B04009	-	A	C	C	-	C	T	-	A	G	T	T	C	A	T	C	A	A

C.

No.	Cis-elements	Sequence	Strand
1	CAATBOX1	CAAT	+
2	**PYRIMIDINEBOXHVEPB1**	TTTTTTCC	-
3	TATABOX5<br>POLASIG1<br>**-300CORE**<br>**300ELEMENT**<br>**EMHVCHORD**	TTATTT<br>AATAAA<br>TGTA(_)AAG<br>TGHA(_)AARK<br>TGTA(_)AAGT	-<br>+<br>+<br>+<br>+
6	GTGANTG10	GTGA	+
7	GT1CONSENSUS<br>GT1GMSCAM4	GRWAAW<br>GAAAAA	-<br>-
10	WBOXATNPR1<br>BIHD1OS<br>WRKY71OS<br>CAATBOX1	TTGAC<br>TGTCA<br>TGAC<br>CAAT	+<br>-<br>+<br>+
12	RAV1AAT<br>**RYREPEATBNNAPA**<br>**GAREAT**	CAACA<br>CATGCA<br>TAACAAR	-<br>-<br>-
