# Supplementary material for: Identification of quantitative trait loci for increased α-tocopherol biosynthesis in wild soybean using a high-density genetic map
Source: BMC Plant Biol. 2019 Nov 21;19:510. doi: 10.1186/s12870-019-2117-z (PMC6873731; doi:10.1186/s12870-019-2117-z)
Supplement: Supplementary file 6 — Additional file 6. The ‘TK780’ × ‘B04009’ linkage map. The map was created based on 7710 SNPs genotypes generated by restriction-site associated DNA sequencing. [file 12870_2019_2117_MOESM6_ESM.pdf]

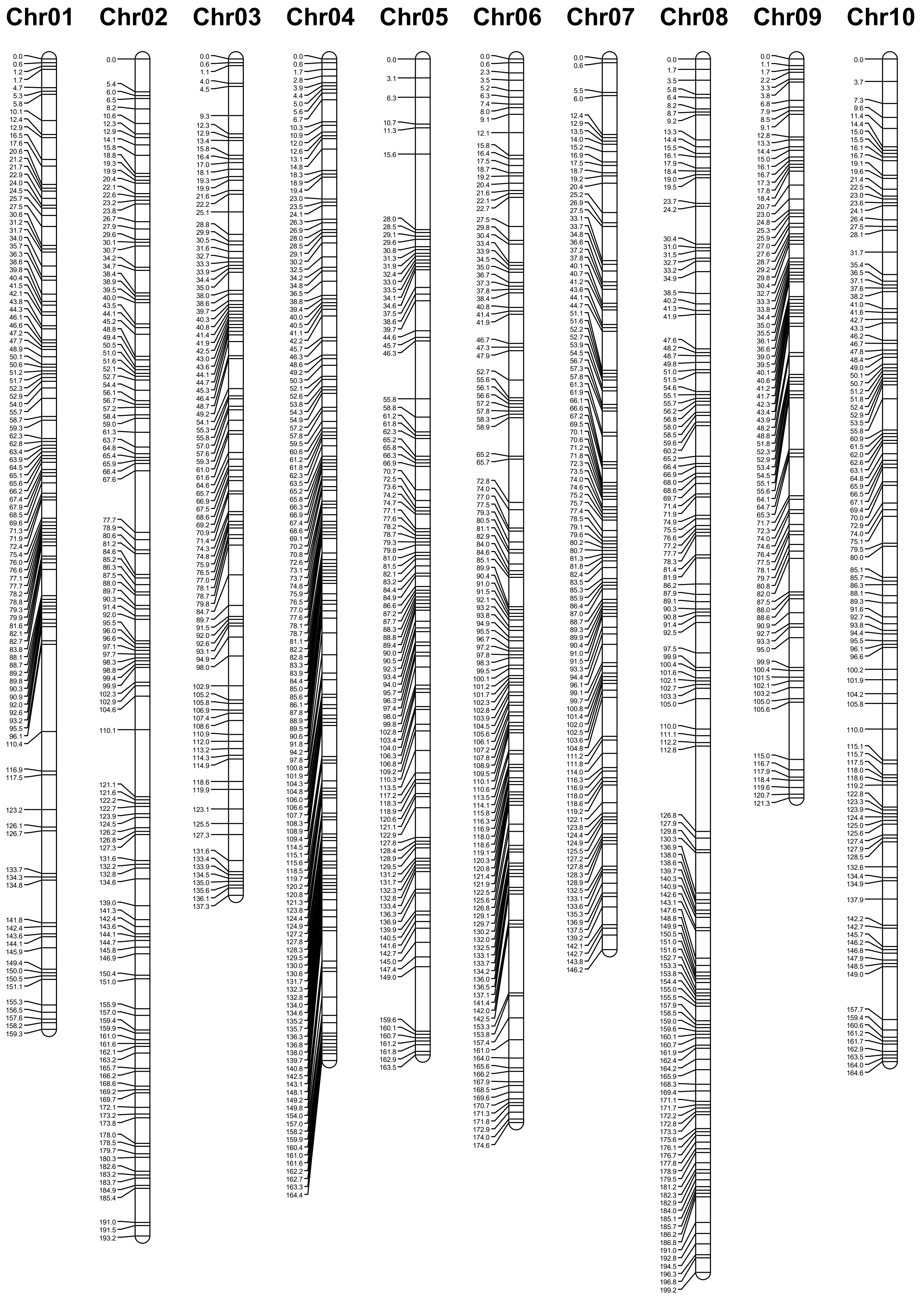
Chr01
Chr02
Chr03
Chr04
Chr05
Chr06
Chr07
Chr08
Chr09
Chr10

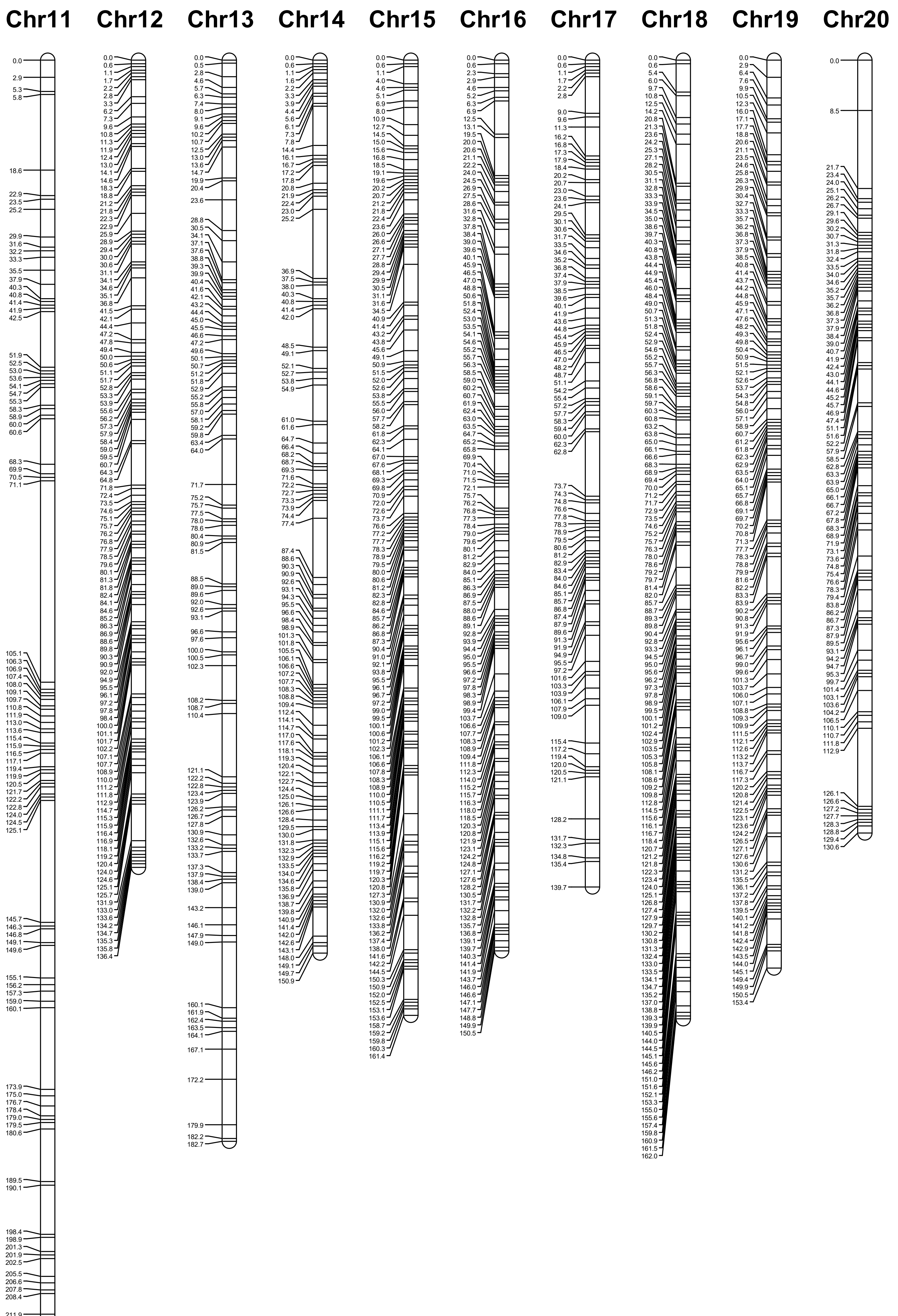
Chr11
Chr12
Chr13
Chr14
Chr15
Chr16
Chr17
Chr18
Chr19
Chr20
